# Energy Coupling of Laser Radiation on AISI 304 Stainless Steel: Effect of High Temperatures and Surface Oxidation

**DOI:** 10.3390/ma12172802

**Published:** 2019-08-30

**Authors:** Dominik Hipp, Achim Mahrle, Eckhard Beyer

**Affiliations:** 1 Institute of Manufacturing Science and Engineering, TU Dresden, PO Box, D-01062 Dresden, Germany; 2 Fraunhofer IWS Dresden, Winterbergstraße 28, D-01277 Dresden, Germany

**Keywords:** energy coupling efficiency, high temperatures, laser material processing, surface oxidation, absorptivity

## Abstract

The industrial application of laser materials processing methods is still far ahead of research into the physical phenomena occurring during these processes. In particular, the effect of high temperatures on the energy coupling of laser irradiation of metals is poorly understood. However, most processes in laser materials treatment involve temperatures above the melting point or even cause evaporation. This study therefore evaluates the effect of high temperatures on the energy coupling efficiency of stainless steel experimentally for three typical laser wavelengths (515 nm, 1.07 µm, 10.6 µm). As a result, it is shown that the effect of temperature on the energy coupling efficiency depends on the wavelength. In this context the relevance of the X-point phenomenon known from the emissivity theory could be demonstrated for laser material processing. Further, the effect of a process-induced surface oxidation is analyzed. At temperatures above 650 °C the energy coupling efficiency dramatically increases to around 65% at melting point and stays at this high level even in the liquid phase.

## 1. Introduction

Nowadays laser materials processing is well-established in industry for several applications such as welding, cutting, cladding, etc. These processes mostly involve temperatures above the melting point and can even reach the boiling point. However, the effect of higher temperatures on the energy coupling of laser radiation is still scarcely explored. Tabulated values of optical properties such as refractive index *n* and extinction coefficient *k* in standard textbooks [1,2,3] are reported for room temperature or for particular measuring conditions far below room temperature. In addition, the absorptivity calculated from the Fresnel equations using these optical properties is only valid for perfect smooth surfaces. Some studies tried to examine the effect of non-laboratory conditions on the energy coupling of laser radiation. While for the effect of surface roughness, both theoretical calculation models [4,5,6] and experimental results [7,8] were published, but the results for the temperature dependence are only sparse. Using Kirchhoff’s law of thermal radiation, which states that under the assumption of a thermodynamic equilibrium the direct spectral emissivity corresponds to the direct spectral absorptivity, results on the temperature dependence of the emissivity can be considered instead. Price [9] ascertained for the emissivity, that the effect of temperature depends on the emitted wavelength of the light. He found, that there is a specific wavelength for each material at which the emissivity is completely independent of the temperature. This wavelength was termed the X-point. At wavelengths above this X-point, the emissivity increases with the temperature while it decreases below. For iron and platinum, the X-point was later found around 2 µm [10] and for aluminum at 0.95 µm [11]. In addition, the X-point was observed in several experiments regarding the temperature dependence of the absorptivity without relating to this phenomenon [8,12,13]. However, one has to distinguish at this point between the absorptivity and the energy coupling efficiency. While the absorptivity is an intrinsic material property, which applies for ideal surfaces and a specific temperature, the energy coupling efficiency is an integral value considering phenomena like multiple reflections due to surface roughness or surface defects on the one hand and a temperature range as a result of the coupled light energy on the other. Therefore, for laser material processing where technical surfaces and temperature ranges from room temperature to melting point are common, the energy coupling efficiency is crucial while the absorptivity only plays a minor role. The afore mentioned X-point is thereby only validated for the influence on the emissivity/absorptivity, the question as to if this phenomenon affects the integral coupling efficiency and consequently has to be considered during laser materials processing is still open. Further, there are contradictory results concerning the energy coupling at temperatures around the phase change of the material. While some studies observed a decrease of the energy coupling efficiency during the CO_2_-laser irradiation of aluminum at higher temperatures [8,14], others report a sudden increase after reaching the liquid phase [15,16,17]. The decrease was explained by possibly structural changes possibly occurring in the probe [14]. A last effect, which is thought to have an influence on the energy coupling efficiency during a real process is the induced surface oxidation. Some studies reported higher values of energy coupling on oxidized surfaces [18,19,20]. Contradictorily, others observed an initial decrease of the coupling efficiency while the effect of oxidation, in turn resulting in higher coupling efficiencies, only takes place above a certain temperature range [21,22,23]. The decreasing coupling efficiency was ruled as due to a cleaning effect of the surface due to the laser irradiation. In [24] the increase in coupling efficiency for stainless steel started above 600 °C.

As one can see, the results in literature contradict themselves in several points. However, the exact knowledge of all the occurring effects is a crucial necessity to understand the processes and therefore be able to optimize them. This study tries to fill this gap by experimentally determining the energy coupling efficiency in dependence on the wavelength, temperature and oxidation state. AISI 304 stainless steel is irradiated with three different laser wavelengths (10.6 µm, 1.07 µm, 0.515 µm) in inert-gas atmosphere as well as for a wavelength of 1.07 µm in an active gas atmosphere. Thus, the X-point phenomenon can be examined and the relevance for laser materials processing can be assessed. In conclusion, the results of this study will provide a knowledge foundation, which will advance our understanding of laser materials processing.

The paper is structured as follows. In Section 2 the measuring method is described as well as the investigated samples are characterized. In Section 3 the experimental results on the temperature dependence and the effect of oxidation are presented and discussed. Section 4 summarizes the results and draws some conclusions.

## 2. Materials and Methods

**Figure 1:**
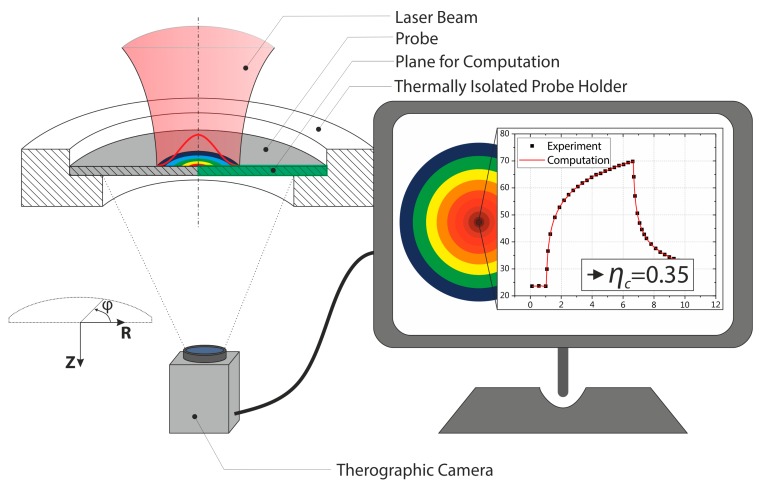
Drawing of the determination method of the temperature dependent coupling efficiency.

Since existing methods for the determination of the absorptivity (e.g., integrated sphere [25] or calorimetry [4]) mostly have shortcomings when it comes to the estimation of the energy coupling at high temperatures, a new measuring technique was developed. This new method is able to determine the coupling efficiency and also to link the results to the process temperature. It consists of two steps. First, the temperature–time curve of the probe irradiation and the subsequent cooling regime was assessed. The energy coupling efficiency was then determined in a second step through the alignment of the experimental temperature–time curves with a heat flow computation. The experimental setup is schematically illustrated in Figure 1 and consisted of a sample holder which thermally isolated the disk-shaped probe at the contact face. The probe was irradiated centrically with a stationary laser beam; the temperature increase of the probe was captured from underneath using a thermographic setup. This allowed to visualize the heat distribution inside the probe, which had to be considered for a high measuring accuracy [26]. For the temperature measurement the emissivity was determined in oven experiments beforehand. The probes were irradiated for 5 s; the temperature signal was recorded for 15 s to also capture the cooling regime.

In the second step, a theoretical temperature–time curve was calculated through a heat flow computation. The experimental setup was designed in a axisymmetric way so that the computation model could be reduced to a 2D problem. Therefore, the temperature distribution was calculated as the solution of the following model equation in cylindrical coordinates *R* and *Z*:

(1) $$\frac{\partial \vartheta }{\partial t}=\frac{1}{\rho c_{\rho }\left(\vartheta \right)}\times \left[\frac{1}{R}\frac{\partial }{\partial R}\left(R\times \lambda_{th}\left(\vartheta \right)\frac{\partial \vartheta }{\partial R}\right)+\frac{\partial }{\partial Z}\left(\lambda_{th}\left(\vartheta \right)\frac{\partial \vartheta }{\partial Z}\right)\right]$$

With *ρ* the density, *c_ρ_*(*ϑ*) the temperature dependent specific heat capacity and *λ_th_*(*ϑ*) the temperature dependent thermal conductivity. Material data were extracted from the database “Total Materia” [27] and are listed in Table A1. The heat flux of the laser beam $\dot{q_{L}}$ as well as the heat losses through convection $\dot{\;q_{C}}$ and thermal radiation $\dot{\;q_{R}}$ were considered as a boundary condition at the top face of the probe (Z = z_top_):

(2) $$\lambda {\left.\frac{\partial \vartheta }{\partial Z}\right|}_{Z=z_{top}}=\;\dot{q_{L}}-\dot{\;q_{C}}-\dot{\;q_{R}}=\frac{2\eta_{c}P_{L}}{\pi R_{0}^{2}}\times \exp\left(-\frac{2\times R^{2}}{R_{0}^{2}}\right)\;-\;h_{t}\left(\vartheta -\vartheta_{0}\right)-\;\varepsilon_{E}\sigma_{B}\left(T^{4}-T_{0}^{4}\right)$$

with *η_c_* the coupling efficiency, *P_L_* the applied laser power, *R_0_* the laser beam radius at the top face of the probe, *h_t_* the heat transfer coefficient, *ε_E_* the emissivity and *σ_B_* the Stefan–Boltzmann constant. The model equations were numerically solved and provided as the result of the computation a theoretical temperature–time curve for the irradiation. It could be shown in a sensitivity analysis (see [28] for details) that the theoretical temperature–time curve only coincides with the experimental curve for one specific value of the energy coupling efficiency. Therefore the model was suited to inversely calculate the energy coupling efficiency. As a further result of the heat flow model, the energy coupling efficiency could be ascribed to the temperatures in the direct interaction zone between laser beam and material during the experiments. This allows the link between the measured coupling efficiency and the prevailing temperature and therefore allows the determination of the temperature dependence of the coupling efficiency.

**Figure 2:**
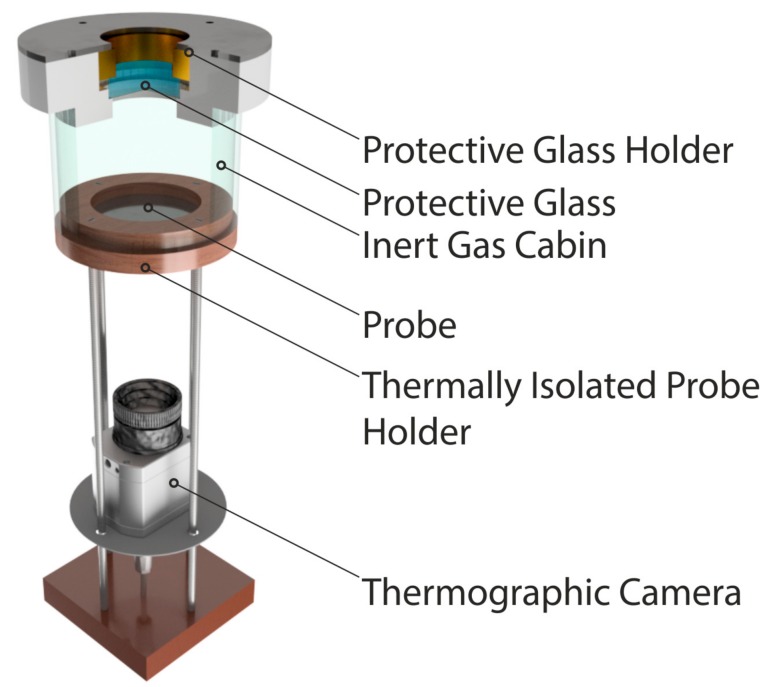
Setup for the determination of the temperature dependent energy coupling efficiency under the usage of a thermographic camera.

The experiments were conducted on AISI 304 stainless steel (X_5_CrNi_18–10_) probes of 60 mm in diameter and 3 mm in thickness. The surface was polished (root mean square surface roughness as deviation from surface normal *R_rms_* = 0.016 µm; spacing between surface roughness profiles *a* = 2.91 µm) to reduce the effect of surface roughness. Three different CW lasers sources were used: a CO_2_-laser (ROFIN-SINAR Laser GmbH, Hamburg, Germany), a fiber laser (IPG Laser GmbH, Burbach, Germany) and a frequency-doubled disk laser (TRUMPF GmbH + Co. KG, Ditzingen, Germany) with wavelengths of 10.6 µm, 1.07 µm and 515 nm, respectively. The process temperature was varied through different optical setups and laser powers (for details see Table A2). The experiments for the evaluation of the temperature dependence were conducted in an argon atmosphere (see inert gas cabin in Figure 2) with a residual oxygen content below 5 ppm. The effect of oxidation was studied for fiber laser irradiation (λ = 1.07 µm) in normal atmosphere. The final experimental setup is depicted in Figure 2. The protective glass was adjusted for the three used wavelengths.

## 3. Results and Discussion

**Figure 3:**
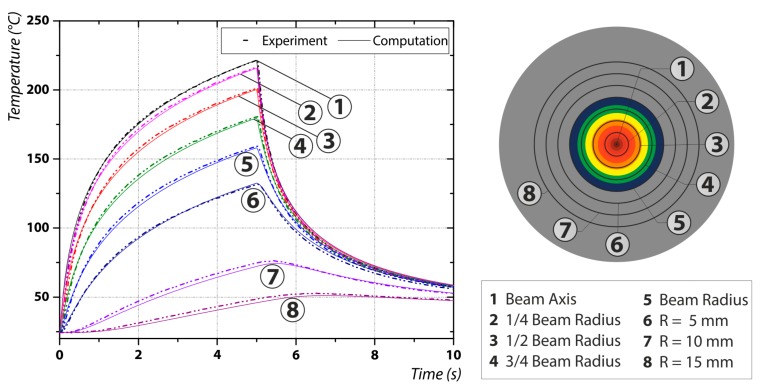
The fit between the computational and experimental temperature–time curves (**left**) at different measuring locations (**right**).

For the evaluation of the energy coupling efficiency, an alignment between experimental and theoretical temperature–time curves is needed. In Figure 3 this is exemplarily demonstrated for one trial run. In can be seen that the fit between experiment and computation is good for both the complete time range and all eight evaluated measuring positions. The deviations at the measuring positions 7 and 8 only evaluate to 2 K and can be neglected. Therefore it can be concluded, that the theoretical model computes the temperature distribution in good agreement to the experiment and can be used to inversely calculate the energy coupling efficiency on the one hand, and the maximum temperature increase through the laser irradiation on the other. Since the temperature drop after turning off the laser at 5 s is mainly influenced by heat conduction inside the probe, the material properties needed for the computation also seem to correspond with the real ones, resulting in a good alignment.

### 3.1. Temperature Dependence

**Figure 4:**
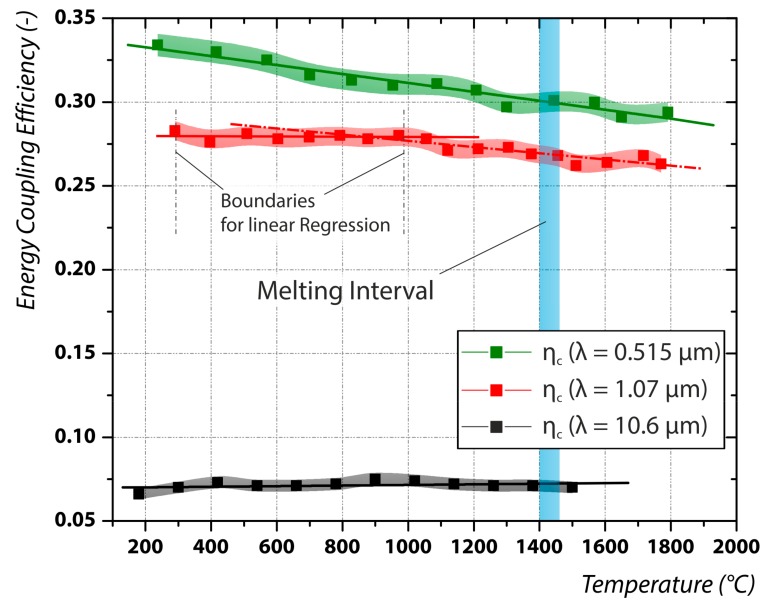
Results for the temperature dependent energy coupling efficiency for the three wavelengths (0.515, 1.07, and 10.6 µm).

The results for the measurement of the temperature dependent coupling efficiency are illustrated in Figure 4. It is obvious that there are different tendencies observable. The energy coupling efficiency of the frequency doubled disk laser (green line) with a wavelength of 0.515 µm monotonically decreases almost linearly with temperature. Also at temperatures above phase change and therefore in the liquid phase the energy coupling efficiency decreases slightly. The decline from *η_c_* = 0.334 at 200 °C to *η_c_* = 0.291 in the liquid phase evaluates to around 13%.

The energy coupling efficiency for laser radiation with 1.07 µm wavelength shows a similar behavior at higher temperatures. While below approximately 1000 °C the coupling efficiency is independent of the temperature, above this temperature a slight decrease of efficiency was measured. The temperature independent energy coupling efficiency is in good agreement with the results for the absorptivity at temperatures below 600 °C in [24]. As mentioned before, the decrease of the absorptivity below the melting point was attributed to a cleaning effect of the surface [21]. In our experiments we believe that a cleaning effect can be excluded for two reasons. First, we used cold rolled probes which were pickled to remove any possibly existing oxidation layer in consequence of the mechanical treatment. The probes were further polished and then cleaned in a ultrasonic ethanol bath. Second, the decrease also continues above the melting point, which contradicts the observations for aluminum [21,22,23]. Therefore, we believe that the measured drop in energy coupling is caused by the temperature increase only.

Interestingly, the energy coupling efficiency of the frequency doubled disk laser (*λ* = 0.515 µm) and the fiber laser (*λ* = 1.07 µm) approximate each other in the liquid phase (Figure 4). Since the energy coupling for light in the visible wavelength spectra is believed to be dominated by interband absorption [29], the measured approach in the liquid phase can be an indication for the theory that in the liquid phase interband absorption is absent [30,31]. Therefore, the believed increased energy coupling efficiency at room temperature for smaller wavelengths [32,33] can diminish in the liquid phase through this effect.

Also, since the energy coupling for both wavelengths (0.515 µm, 1.07 µm) shows a negative temperature coefficient, this indicates that the X-point for 304 stainless steel is located in the mid-infrared. The results for the energy coupling efficiency for CO_2_-laser radiation also confirm this hypothesis. For a wavelength of 10.6 µm the energy coupling efficiency is completely independent of the underlying process temperature. The measured trend in our experiments is identical compared to results in the literature [24], however, our values are lower. This can be a consequence of the much smaller surface roughness [7] of our used probes.

Further, for all investigated wavelengths no sudden change of the coupling efficiency at the phase change was detected. Therefore, contradictory to literature, it can be stated that for polished stainless steel probes a phase change does not affect the energy coupling at all investigated wavelengths. Thus, the sudden change at the phase transition observed in the literature might be an effect of a surface smoothening occurring in the liquid phase [34], however, this thesis must be further investigated.

### 3.2. Effect of Surface Oxidation

**Figure 5:**
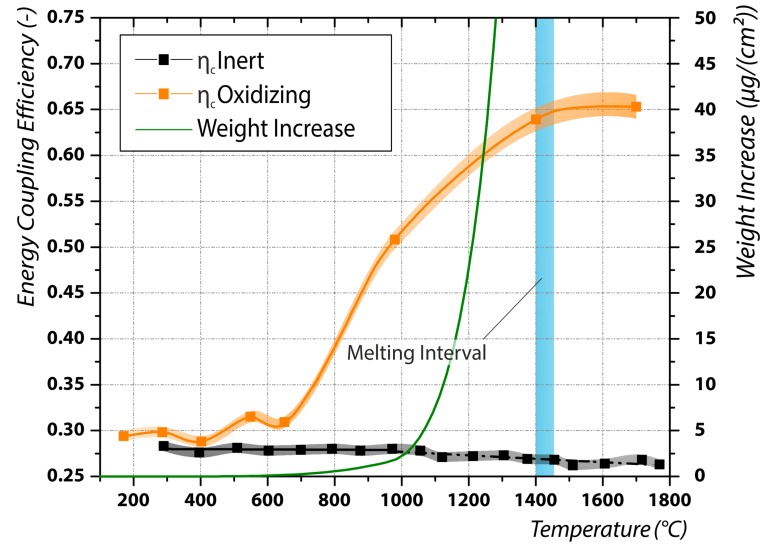
Results for the evaluation of the effect of a surface oxidation on the temperature dependent energy coupling efficiency for fiber laser radiation (*λ* = 1.07 µm).

In Figure 5 the measured coupling efficiency for fiber laser radiation (*λ* = 1.07 µm) in an oxidizing environment is compared to the one in an inert gas atmosphere. Below 600 °C the agreement between the two curves is good. Above around 650 °C the energy coupling efficiency in the oxidizing environment increases dramatically, reaching a value of *η_c_* = 0.508 at 1000 °C and *η_c_* = 0.653 in the liquid phase. Interestingly, the energy coupling efficiency below and above the phase transition are comparable, although the solidified melt pool clearly shows signs of a melt pool convection. One reason for this may be found in the high melting point of chromium-III-oxide (2329 °C), which is preferably formed during laser irradiation of stainless steel due to the high oxygen affinity of chromium [35]. Therefore it is believed, that the chromium-III-oxide is still in the solid phase and covers the melt pool. Chromium-III-oxide exhibits a high absorptivity of 81.9% [36], wherefore the measured energy coupling efficiency is high. However, it also exhibits a high attenuation length of 4400 nm and is thus partly transparent for the fiber laser radiation. The measured energy coupling efficiency is therefore believed to be a mix of the energy coupling of the oxide layer and of the base metal beneath.

By using data of [37] and applying these to the Arrhenius law and parabolic rate law, the weight increase through the oxidation can be theoretically calculated. This is also illustrated in Figure 5. It is obvious that the weight increase coincides with the increase in coupling efficiency at temperatures above 650 °C. Therefore, the measured higher coupling efficiency can be directly related to the oxidation process. Further, by using these data, a temperature dependent oxide layer thickness can be calculated. At melting point this evaluates to around 600 nm in thickness. Using the Beer–Lambert law, the calculated attenuation of the laser irradiation by the oxide layer (Cr_2_O_3_) is evaluated at the melting point to be 40%. This is again an indication for the thesis above, that the measured energy coupling efficiency is a mix of the coupling in the oxide layer and in the base metal below.

The measured temperature threshold (650 °C) for the increase in coupling efficiency as a consequence of a process-induced oxidation in Figure 5 is in good agreement with data provided in the literature [24]. However, we measured a steeper increase of the coupling efficiency. Further our experiments demonstrated that the increase continues up to the melting point and remains on this high level even in the liquid phase.

## 4. Summary and Conclusions

The knowledge of the physical phenomena impacting the energy coupling efficiency during laser material treatment is a fundamental necessity to assess, understand and optimize these processes. However, little is known about the effect of high temperatures as well as of a process-induced surface oxidation on the energy coupling of a laser beam in materials. This study experimentally evaluated the effects of high temperatures and of an oxidizing environment on the energy coupling efficiency on stainless steel. The following conclusions can be drawn:

The energy coupling mechanism of laser radiation at high temperatures depends on the wavelength.
The energy coupling efficiency of visible laser irradiation (*λ* = 0.515 µm) shows an approximately linear decrease with temperature.
The energy coupling efficiency for laser irradiation in the near infrared (*λ* = 1.07 µm) is below 1000 °C independent of the temperature, above a linear decrease was measured with temperature.
The energy coupling efficiency for CO_2_ laser irradiation (*λ* = 10.6 µm) is completely independent of the temperature for the evaluated temperature range.
The process-induced oxidation leads to a dramatic increase of the energy coupling efficiency above 650 °C and stays at this high level also in the liquid phase.

The experimental results are of special interest for the process understanding in the field of laser material processing. It could be shown that the X-point phenomenon known from emissivity and absorptivity theory also influences the integral value of the energy coupling efficiency. Thus, the relevance of the X-point for laser material processing could be demonstrated. As a result, the advantage of increased energy coupling at room temperature for laser wavelengths in the visible range compared to near infrared diminishes in the liquid phase for the evaluated stainless steel.

## Appendix A

**Table A1:** Material data AISI 304.

Density (kg/m^3^)	293 K	8030
Heat conductivity (W/(mK))	293 K	14.8
	400 K	16.6
	500 K	18.3
	600 K	19.8
	700 K	21.2
	800 K	22.6
	900 K	24
	1000 K	25.4
	1100 K	25.7
Heat capacity (J/(kg·K))	293 K	500
	473 K	510
	673 K	550
	873 K	585
	1073 K	630

**Table A2:** Laser beam data.

Header	Wavelenth *λ* (µm)	Beam Radius (mm)	Laser Power (W)
Frequency-doubled disk laser	0.515	3.68	0–1000
Fiber laser	1.07	0.486	0–400
CO_2_ laser	10.6	3	0–2400
